# Risk Factors of Regional Lymph Node Metastasis in Patients with Cervical Cancer

**DOI:** 10.1515/biol-2019-0023

**Published:** 2019-06-24

**Authors:** Chunchun Wu, Lichun Li, Xue Xiao, Anyi Sun, Wenji Lin, Ailu Li

**Affiliations:** 1 No. 248 Dong Road Quanzhou City Fujian Province 362000 PR China; 2 Department of Obstetrics and Gynecology, Quanzhou First Hospital Affiliated to Fujian Medical University, Quanzhou Fujian Province 362000 PR China; 3 Department of Obstetrics and Gynecology, Medical College of Nanchang University, 330031 Nanchang PR China; 4 Department of Ultrasonography, Quanzhou First Hospital Affiliated to Fujian Medical University, Quanzhou Fujian Province 362000 PR China; 5 Department of Radiology, Quanzhou First Hospital Affiliated to Fujian Medical University, Quanzhou Fujian Province 362000 PR China

**Keywords:** cervical cancer, lymph node metastasis, risk factors, logistic regression, prognosis

## Abstract

**Objectives:**

To explore the risk factors related to regional lymph node metastasis in cervical cancer and analyze the value of independent risk factors in predicting regional lymph node metastasis.

**Methods:**

We retrospectively analyzed the clinical data of 699 patients who underwent surgery for stage IB1–IIA2 cervical cancer in Quanzhou First Hospital affiliated to Fujian Medical University from 2010 to 2016. The patients were divided into metastasis (*n* = 92) and non-metastasis (*n* = 607) groups based on the postoperative pathology of regional lymph node status. The relevant clinicopathological features of the metastasis and non-metastasis groups were compared through variance analysis and chi-square tests. Logistic regression was adopted to screen relevant independent risk factors of regional lymph node metastasis.

**Results:**

In univariate analysis, International Federation of Gynecology and Obstetrics (FIGO) stages, serum squamous cell carcinoma antigen (SCC-Ag), histological type of squamous carcinoma and maximal tumor diameter were related factors for lymphatic metastasis in patients with cervical cancer. In multivariate analysis, SCC-Ag and histological type of squamous carcinoma were independent prognostic factors for lymphatic metastasis in patients with cervical cancer. Pre-treatment SCC-Ag serum levels, as a predictor of lymph node metastasis of cervical cancer, revealed a sensitivity of 62.07% (95% confidence interval (CI): 51.03–72.62%), specificity of 65.15% (59.07–70.89%), and area under the receiver operating characteristic (ROC) curve of 0.69 (95% CI: 0.61–0.76).

**Conclusions:**

Cervical cancer patients whose pathological type is squamous carcinoma with high levels of SSC-Ag pre-operation are more likely to be diagnosed with regional lymph node metastasis. Standardized lymph node dissection should be implemented during operation.

## 1 Introduction

At present, the standard operation for stage IB1–IIA2 cervical cancer is radical hysterectomy and pelvic lymphadenectomy with improved 5-year survival rate after operation [1]. Most patients are found to be with negative lymph nodes after postoperative pathology, and an early clinical stage means a low probability of developing lymph node metastasis [2]. Disputes on whether the lymph node should be resected for cervical cancer patients, especially those with locally advanced cervical cancer, are common [3, 4, 5]. If lymph node metastasis can be accurately predicted before surgery, lymph node-negative patients can avoid unnecessary lymph node dissection and the prognosis of such patients may be improved. However, a perfect biological model that can be used to predict regional lymph node metastasis of cervical cancer before operation is unavailable yet. Therefore, we conducted a retrospective analysis of data on patients who underwent surgery for IB1–IIA2 cervical cancer in Quanzhou First Hospital affiliated to Fujian Medical University from 2010 to 2016. Logistic regression was adopted to screen the independent risk factors of lymph node metastasis of cervical cancer. The risk factors obtained may serve as a useful reference for exploring and predicting the clinical value of lymph node metastasis.

## 2 Materials and methods

### 2.1 Patients

**Figure 1:**
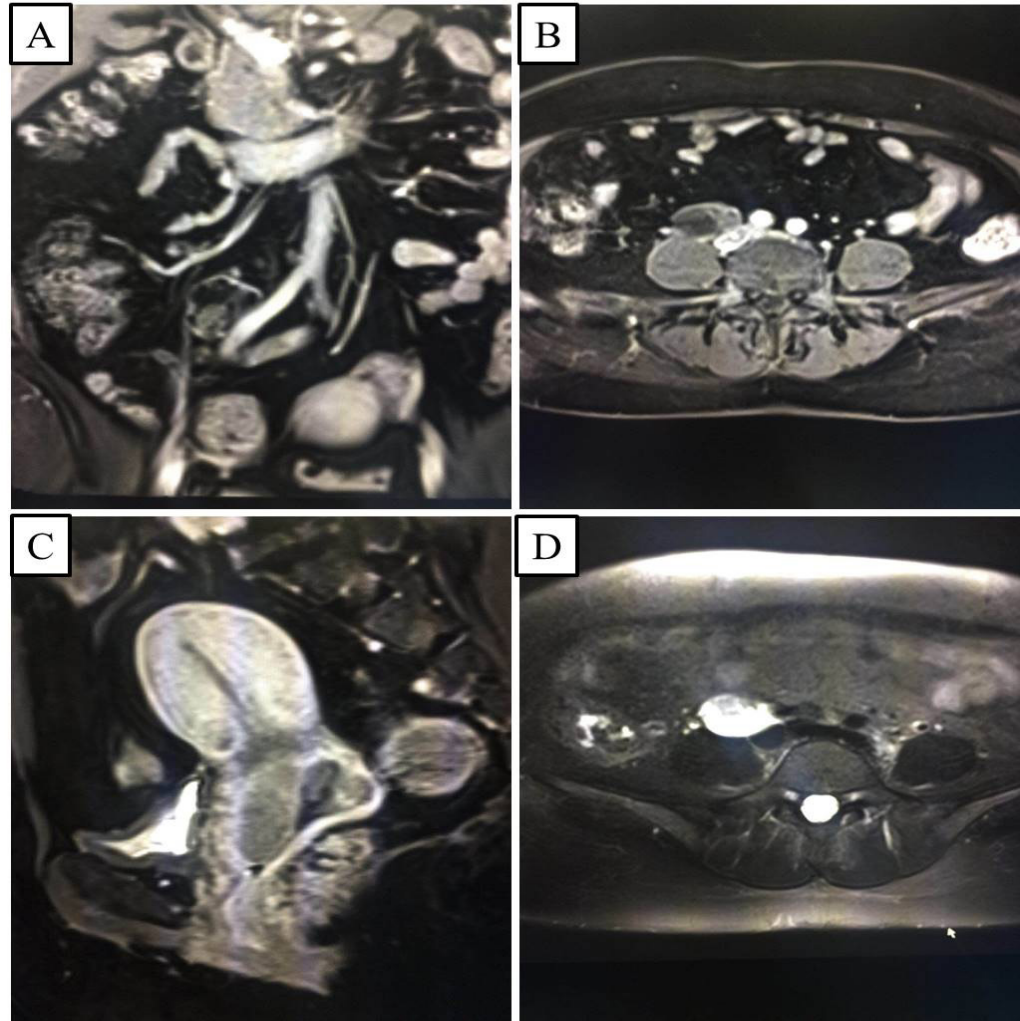
Magnetic resonance imaging (MRI) of cervical cancer and local regional metastatic lymph nodes. A: Coronal view of MRI showing lymph node metastases to the right iliac artery and uneven enhancement. B: Axis view enhancement showing the location of metastatic lymph nodes. C: Sagittal view of cervical cancer. D: Axis view t2-weighted imaging (T2WI) showed the right iliac artery para metastatic lymph node, partial liquefaction and necrosis.

We performed a retrospective analysis of the clinical data of 699 patients who underwent IB1–IIA2 cervical cancer surgery in Quanzhou First Hospital affiliated to Fujian Medical University from 2010 to 2016 (**Figure 1**). The patients were divided into the metastasis group (*n* = 92) and the non-metastasis group (*n* = 607) based on the postoperative pathology examination. The inclusion criteria of the patients were as follows: (1) primary tumor occurring in the cervix with a clear pathological diagnosis; (2) details of the patient’s clinical baseline conditions, such as age, medical records, previous treatment, were complete; (3) clinical stage of IB1–IIA2; (4) patient underwent radical hysterectomy and pelvic lymphadenectomy; and (5) no distant metastasis of the lung, liver, and abdomen during preoperative examination. The exclusion criteria of the patients were: (1) incomplete medical record; (2) no implementation of lymph node dissection; (3) peritoneal dissemination of the tumor or distant metastasis; and (4) no implementation of pelvic magnetic resonance imaging (MRI) inspection before operation.

**Ethical approval**: The research related to human use has been complied with all the relevant national regulations, institutional policies and in accordance the tenets of the Helsinki Declaration, and has been approved by the Medical Ethics Committee of Quanzhou First Hospital’s.

### 2.2 Clinical data collection

Two researchers independently included medical records according to the inclusion and exclusion criteria. Medical records under dispute were discussed by the two researchers to decide whether the data could be included in the study. The excerpted clinical data of patients included age, menopause history, pregnancy history, clinical stage, pelvic MRI results, pathological data (pathological type, differentiation, and maximum tumor diameter), and whether neoadjuvant chemoradiotherapy was applied before operation. The clinical database was cross-checked by the two researchers.

### 2.3 Research and quality control

Selective bias control: To reduce or prevent inclusion bias arising from the inclusion process of medical records, we trained the two personnel responsible for accepting the medical records based on the inclusion and exclusion criteria and excerpting information by using a 10-point standard. Bias measurement control: a uniform scale was used for data inputs, and different diagnoses or evaluation standards of cases were converted. Confounding bias control: single-factor chi-square inspection was applied to screen factors that may be relevant to regional lymph node metastasis. Possible factors were then input into a logistic regression equation to calculate independent risk factors and remove confounding factors.

### 2.4 Statistical analysis

Statistical Package for Social Sciences17.0 software (SPSS, Inc., Chicago, IL, USA) was used for data analysis. The measurement data was expressed with *x* ± *s* and the comparison between groups was made based on the t-test of the sample mean. The enumeration data were shown with a relative number, and the comparison between groups was made based on the c2 or Fisher’s exact test. Univariate analysis was performed for each candidate variable and P<0.05 was considered to indicate a statistically significant difference. Logistic regression analysis was conducted to identify which factors were associated with the prognosis. Receiver operating characteristic curve (ROC) analysis was performed to assess serum squamous cell carcinoma antigen (SCC-Ag) concentration as a biomarker for local regional lymph node metastasis. As in the previous analysis, P<0.05 indicated a statistical difference.

## 3 Results

### 3.1 Clinicopathological features of the metastasis and non-metastasis groups

Data in the cervical cancer lymph node metastasis and non-metastasis groups differed in terms of International Federation of Gynecology and Obstetrics (FIGO) stage (p < 0.05), serum concentration of SCC-Ag (p < 0.05), histological type (p < 0.05), and maximum tumor diameter (p < 0.05), see Table 1.

**Table 1:** General characteristics of the two groups, metastasis and non-metastasis, of patients under treatment for cervical cancer (2010–2016).

Characteristics			P value

	Non-metastasis (n=607)	Metastasis (n=92)	
Age (year)	46.98±9.71	45.22±8.12	0.099
Education [n]			0.722
Illiteracy or primary school	285	46	
Junior middle school	218	36	
Senior high school	74	10	
College	28	2	
Height (cm)	159.08±6.06	158.83±3.06	0.928
Weight (kg)	61.14±9.40	62.48±8.96	0.227
Gravidity	3.79±1.93	3.64±1.78	0.499
Parity	2.42±1.30	2.23±1.05	0.188
FIGO stage			0.013
IB1	356	39	
IB2	103	24	
IIA1	94	15	
IIA2	54	14	
Serum SCC-Ag (μg/L)	3.01±4.98	7.10±11.21	<0.001
Pathology type			0.002
Non-squamous cell carcinoma	107	4	
Squamous cell carcinoma	500	88	
Tumor gross type			0.51
Exogenic	391	56	
Endogenetic	197	29	
Ulcerative	15	5	
Others	5	1	
Tumor differentiation			0.578
Well	21	0	
Well to Moderate	29	6	
Moderate	271	38	
Moderate to Poor	154	29	
Poor	126	25	
Tumor diameter (cm)	3.56±1.34	4.05±1.46	0.001
Neoadjuvant chemoradiotherapy			0.068
Negative	503	69	
Positive	104	23	

FIGO : International Federation of Gynecology and Obstetrics; SCC-Ag: squamous cell carcinoma antigen

### 3.2 Analysis of relevant pre-treatment factors influencing lymph node metastasis

Single-factor analysis showed that FIGO stage, serum SCC-Ag, histological type, and maximum tumor diameter were related to lymph node metastasis of cervical cancer. Logistic regression analysis showed that serum SCC-Ag >2.5 μg/L [OR = 1.072 (1.028–1.119)] before treatment and histological type of squamous cell carcinoma [OR = 4.147 (1.251–13.742)] were independent risk factors of lymph node metastasis of cervical cancer, see Table 2.

**Table 2:** Risk factors relevant to local regional lymph node metastasis by logistic regression in patients under treatment for cervical cancer (2010–2016).

Characteristics	Single factor analysis		Multifactor analysis	

	OR (95% CI)	p	OR (95% CI)	p
Age	0.980 (0.957-1.004)	0.100	NA	NA
Education	0.898 (0.628-1.181)	0.441	NA	NA
Height	0.991 (0.818–1.199)	0.922	NA	NA
Weight	1.016 (0.990–1.042)	0.225	NA	NA
Gravidity	0.958 (0.846–1.085)	0.497	NA	NA
Parity	0.874 (0.717–1.066)	0.184	NA	NA
FIGO stage	1.374 (1.131–1.669)	0.001	1.036 (0.758–1.415)	0.825
Serum SCC-Ag	2.255 (1.328–3.828)	^＜^0.001	1.072 (1.028–1.119)	0.001
Squamous cell carcinoma	4.708 (1.692–13.102)	0.012	4.147 (1.251–13.742)	0.020
Tumor differentiation	1.220 (0.896–1.662)	0.207		
Tumor diameter	1.299 (1.107–1.524)	0.001	1.137 (0.874–1.479)	0.337

FIGO: International Federation of Gynecology and Obstetrics; SCC-Ag: squamous cell carcinoma antigen; OR: odds ratio; CI: confidence interval; NA: not applicable

### 3.3 Value of pre-treatment serum SCC-Ag levels in predicting cervical cancer lymph node metastasis

**Figure 2:**
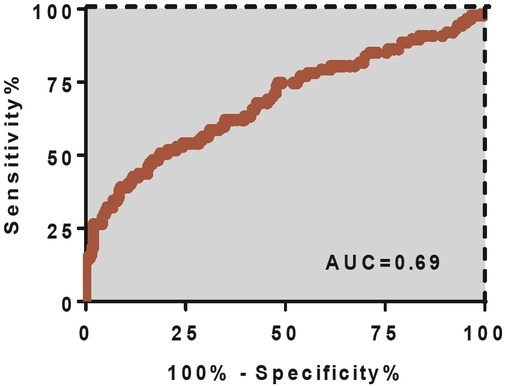
Receiver operating characteristic (ROC) curve of serum squamous cell carcinoma antigen (SCC-Ag) as a biomarker for local regional lymph node metastasis in cervical cancer patients. Area under the curve (AUC) = 0.69.

The use of pre-treatment SCC-Ag serum concentration as a predictor of lymph node metastasis of cervical cancer reveals a sensitivity of 62.07% (95% CI: 51.03–72.62%), specificity of 65.15% (59.07–70.89%), and area under the receiver operating characteristic (ROC) curve of 0.69 (95% CI: 0.61–0.76), see **Figure 2**.

## 4 Discussion

Clinical stage is the most important factor influencing cervical cancer prognosis [5, 6, 7]. The major route of cervical cancer metastasis is lymph node metastasis. Lymph node metastasis poses a considerable influence on cervical cancer prognosis. Radical hysterectomy and pelvic lymph node lymphadenectomy are effective treatment modalities for cervical cancer. When lymph node metastasis is found in the postoperative pathology examination, radiochemotherapy is added to the treatment plan to decrease the recurrence and mortality rate of early cervical cancer. However, the 5-year survival rate of pelvic lymph

node metastasis patients is still considerably lower than that of patients with non-pelvic lymph node metastasis [2]. Therefore, different views exist regarding whether pelvic lymph node lymphadenectomy is needed for successful cervical cancer treatment. Some doctors do not perform pelvic lymph node lymphadenectomy because of difficulties associated with completely dissecting the lymph node, especially since most pelvic lymph node-positive patients show metastasis near the abdominal aorta. Under such circumstances, the benefit–risk ratio

of these patients is low. About 80–90% of cervical cancer patients do not experience pelvic lymph node metastasis. The results of a multi-center random clinical study showed that, when technological conditions permit, surgical treatment for locally advanced cervical cancer patients was safe [8]. Therefore, prediction of lymph node metastasis before treatment plays an important role in guiding individual treatment and prompting patient prognosis.

Researchers have discovered that lymph vascular space invasion, invasion depth of the cervical matrix, maximum tumor diameter, clinical stage, and serum SCC-Ag concentrations are related to lymph node metastasis [9, 10]. Our results showed that the pre-treatment factors influencing lymph node metastasis include clinical stage, serum SCC-Ag concentration, histological type of squamous carcinoma, and maximum tumor diameter. Among these factors, serum SCC-Ag concentration and histological type of squamous carcinoma are independent risk factors of lymph node metastasis. Current research indicates that the lymph node metastasis rates of the IB and IIA stages are about 16% and 19%, respectively[10]. Single-factor analysis showed that stage and maximum tumor diameter are risk factors of lymph node metastasis but not the independent risk factors, which is consistent with the research results of Zhao Deying et al. [11]. This finding indicates that lymph node metastasis is caused by cervical cancer of the IB–IIA stages and maximum tumor diameter through high level of serum SCC-Ag, in agreement with the conclusions of Ohara [12] and Kim

[13], who associated serum SCC-Ag expression with tumor size.

SCC-Ag is a subtype of tumor associated antigen TA-4. Normal squamous cells produce low levels of SCC-Ag, whereas cancerous squamous cells produce high levels of the antigen, making the antigen an important marker of cervical cancer [14]. Research shows that, as SCC-Ag serum concentrations increase, the FIGO stages of cervical cancer, maximum tumor diameter, and matrix invasion depth become more associated with lymph node metastasis [13, 15, 16]. The metastasis route of cervical cancer is mainly lymphatic metastasis; hematogenous metastasis is rarely seen. The most common type of cervical cancer is squamous carcinoma, which is consistent with a previous study [17] that found that squamous carcinoma mainly occurs through lymphatic metastasis whereas adenocarcinoma mainly occurs through hematogenous metastasis. Therefore, the present work finds that serum SCC-Ag concentration and histological type of squamous carcinoma are independent risk factors influencing lymph node metastasis.

Pre-treatment serum SCC-Ag concentrations can be used to predict lymph node metastasis but with low sensitivity and specificity. Given that the current work only considers pre-treatment clinical factors in analyzing the relevant factors influencing lymph node metastasis, the diagnostic value of lymph node metastasis could be of less importance than postoperative pathology and other factors. Finally, the results present some significance in predicting the lymph node metastasis condition before treatment, selecting the operation mode, and influencing the decision to complete standardized lymph node dissection.
